# Common Pathogenic Effects of Missense Mutations in the P-Type ATPase ATP13A2 (*PARK9*) Associated with Early-Onset Parkinsonism

**DOI:** 10.1371/journal.pone.0039942

**Published:** 2012-06-29

**Authors:** Agata Podhajska, Alessandra Musso, Alzbeta Trancikova, Klodjan Stafa, Roger Moser, Sarah Sonnay, Liliane Glauser, Darren J. Moore

**Affiliations:** Laboratory of Molecular Neurodegenerative Research, Brain Mind Institute, School of Life Sciences, Ecole Polytechnique Fédérale de Lausanne (EPFL), Lausanne, Switzerland; University of Pittsburgh, United States of America

## Abstract

Mutations in the *ATP13A2* gene (PARK9) cause autosomal recessive, juvenile-onset Kufor-Rakeb syndrome (KRS), a neurodegenerative disease characterized by parkinsonism. KRS mutations produce truncated forms of ATP13A2 with impaired protein stability resulting in a loss-of-function. Recently, homozygous and heterozygous missense mutations in *ATP13A2* have been identified in subjects with early-onset parkinsonism. The mechanism(s) by which missense mutations potentially cause parkinsonism are not understood at present. Here, we demonstrate that homozygous F182L, G504R and G877R missense mutations commonly impair the protein stability of ATP13A2 leading to its enhanced degradation by the proteasome. ATP13A2 normally localizes to endosomal and lysosomal membranes in neurons and the F182L and G504R mutations disrupt this vesicular localization and promote the mislocalization of ATP13A2 to the endoplasmic reticulum. Heterozygous T12M, G533R and A746T mutations do not obviously alter protein stability or subcellular localization but instead impair the ATPase activity of microsomal ATP13A2 whereas homozygous missense mutations disrupt the microsomal localization of ATP13A2. The overexpression of ATP13A2 missense mutants in SH-SY5Y neural cells does not compromise cellular viability suggesting that these mutant proteins lack intrinsic toxicity. However, the overexpression of wild-type ATP13A2 may impair neuronal integrity as it causes a trend of reduced neurite outgrowth of primary cortical neurons, whereas the majority of disease-associated missense mutations lack this ability. Finally, ATP13A2 overexpression sensitizes cortical neurons to neurite shortening induced by exposure to cadmium or nickel ions, supporting a functional interaction between ATP13A2 and heavy metals in post-mitotic neurons, whereas missense mutations influence this sensitizing effect. Collectively, our study provides support for common loss-of-function effects of homozygous and heterozygous missense mutations in *ATP13A2* associated with early-onset forms of parkinsonism.

## Introduction

In recent years, a number of genes have been identified that are associated with autosomal recessive forms of parkinsonism including *parkin (PARK2)*, *DJ-1 (PARK7)*, *PINK1 (PARK6)* and *ATP13A2 (PARK9)* [1], [2], [3]. Mutations in the *ATP13A2* gene cause Kufor-Rakeb syndrome (KRS), a juvenile-onset pallido-pyramidal neurodegenerative disorder characterized by slowly progressive levodopa-responsive parkinsonism often with additional features including supranuclear gaze palsy, pyramidal dysfunction, dystonia and dementia [4], [5], [6]. KRS subjects normally exhibit generalized brain atrophy with evidence of impaired nigrostriatal dopaminergic function [7], [8], [9], [10]. Homozygous or compound heterozygous mutations have been identified in KRS subjects of families from Jordan, Chile, Afghanistan, Pakistan and China that produce frameshift or splicing variants resulting in truncated forms of ATP13A2 protein that are predicted to lead to a loss-of-function [5], [8], [11], [12]. Recent studies have shown that such truncating mutations promote the mislocalization of ATP13A2 to the endoplasmic reticulum (ER) in mammalian cells where they are degraded by the proteasome via the ER-associated degradation (ERAD) pathway [5], [11], [13]. A number of homozygous (F182L [Japan] [14], G504R [Brazil] [10] and G877R [Italy] [9]) and heterozygous (T12M [Italy] [10], G533R [Italy] [10] and A746T [Taiwan/Singapore] [15]) missense mutations have recently been identified in subjects with early-onset forms of familial or sporadic parkinsonism or Parkinson's disease (PD) suggesting that *ATP13A2* mutations may also contribute to early-onset PD. Of interest, homozygous mutations (F182L, G504R and G877R) typically cause juvenile-onset parkinsonism (10 to 22 years) whereas heterozygous mutations (T12M, G533R and A746T) are associated with early-onset parkinsonism (<50 years) [9], [10], [14], [15], potentially suggesting a gene dosage or graded effect of mutations. In contrast to truncating KRS mutations, the mechanism by which missense mutations cause parkinsonism or PD is unclear at present.

The human *ATP13A2* gene encodes an 1180 amino acid protein of ∼130 kDa belonging to the P_5_ subfamily of P-type transport ATPases that are predicted to contain ten transmembrane-spanning domains [16]. Disease-associated missense mutations are located within the intracellular and extracellular regions of the protein with a particular cluster in the second intracellular loop region containing the catalytic ATPase domain. The physiological function of ATP13A2 in mammalian cells is unknown. ATP13A2 is highly expressed in the mammalian brain with particular enrichment in the substantia nigra [5]. In PD brains, ATP13A2 expression is up-regulated in surviving substantia nigra dopaminergic neurons [5], [17]. ATP13A2 is localized at least in part to lysosomal membranes in mammalian cells where it is predicted to participate in the active transport of cations across vesicular membranes in an ATP-dependent manner [5], [16]. However, at present, the cation transporting activity or selectivity of ATP13A2 has not been directly demonstrated. In yeast, deletion of the ATP13A2 ortholog, *ykp9* (*YOR291W*), confers sensitivity to growth in the presence of heavy metals, including cadmium (Cd^2+^), manganese (Mn^2+^), nickel (Ni^2+^) and selenium (Se^2+^) [18], [19], implicating Ykp9 in sequestering heavy metals. ATP13A2 orthologs have been reported to protect against cellular toxicity induced by expression of the PD-associated protein α-synuclein in yeast, nematode worm and primary midbrain dopaminergic neurons [18]. KRS mutations produce unstable truncated forms of ATP13A2 but are also directly toxic to cells, induce ER stress and sensitize cells to ER stress-induced cell death [5], [11], [13]. Thus, truncating KRS mutations most likely cause disease through a loss-of-function mechanism but they may also induce additional cellular toxicity.

It is not yet clear how homozygous missense mutations (F182L, G504R and G877R) in ATP13A2 associated with parkinsonism potentially cause disease as they are likely to have subtle effects compared to truncations [9], [10], [14]. At this juncture it is not clear whether heterozygous mutations in ATP13A2 (T12M, G533R and A746T) are truly disease-causing since they could represent rare polymorphic variants, or pathogenic variants that contribute to disease in combination with unidentified mutations, in a dominant or haploinsufficient manner, or as risk factors [10], [15]. A complete understanding of the effects of disease-associated missense mutations on the basic properties of ATP13A2 will help to clarify their mechanism of action and whether they are likely to represent authentic pathogenic variants. Here, we comprehensively examine the effects of parkinsonism-associated homozygous and heterozygous missense mutations on the protein stability, subcellular localization and ATPase activity of ATP13A2, and their effects on neuronal integrity. Our data demonstrate that homozygous mutations commonly impair the protein stability of ATP13A2 and lead to its enhanced proteasomal degradation, whereas heterozygous mutations commonly impair the ATPase activity of ATP13A2. We further demonstrate that overexpression of wild-type ATP13A2 impairs neurite outgrowth whereas the majority of heterozygous and homozygous missense mutations lack this ability. Finally, the overexpression of ATP13A2 sensitizes to neurite shortening induced by exposure to the heavy metal ions, cadmium or nickel, and missense mutations influence this effect. Our data support a common loss-of-function mechanism for missense mutations associated with early-onset parkinsonism.

## Results

### Disease-associated missense mutations reduce the steady-state levels of ATP13A2

**Figure 1:**
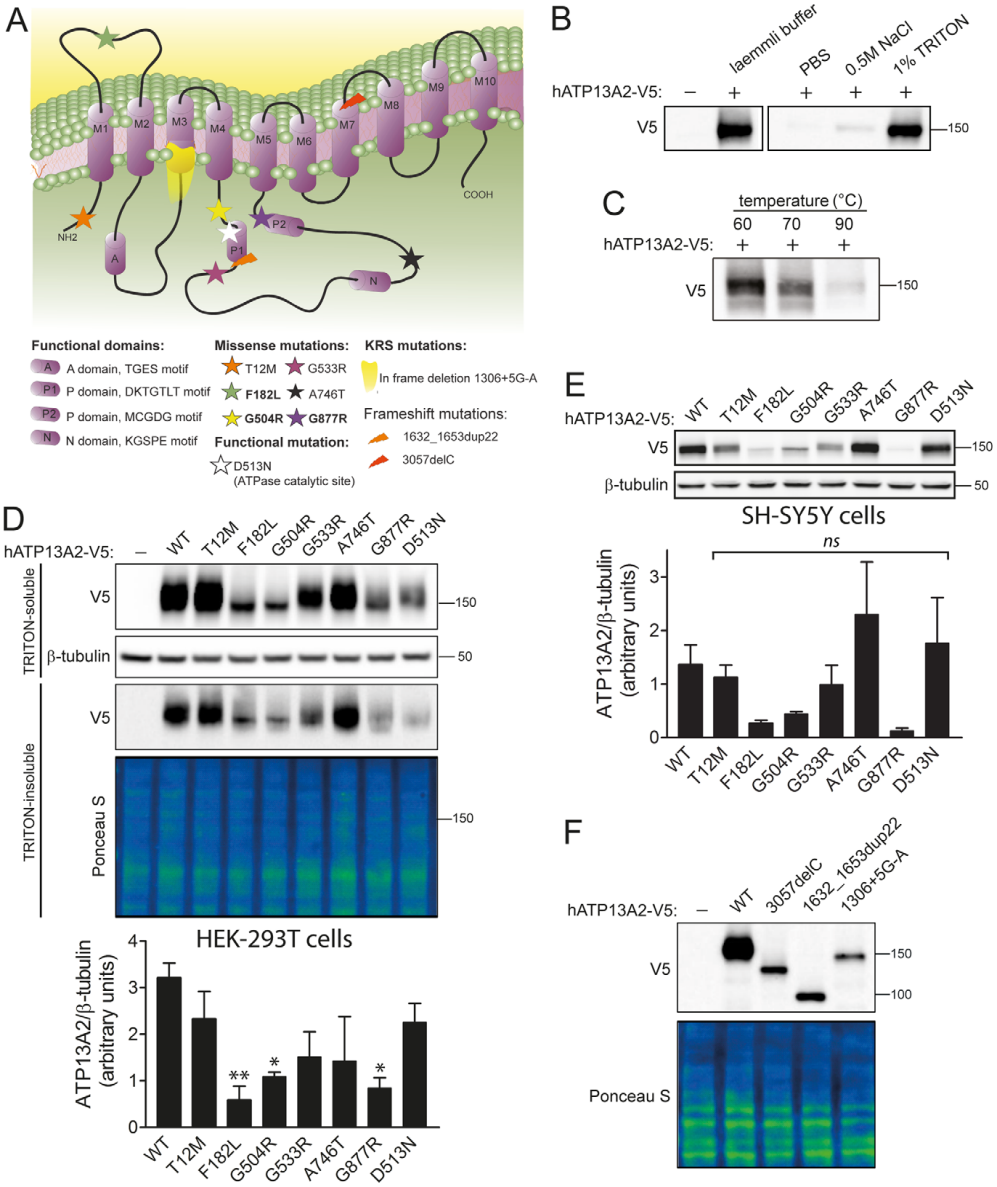
Disease-associated mutations reduce the steady-state levels of ATP13A2 protein. *A* , Predicted domain topology model of human ATP13A2. Wild-type (WT) protein consists of ten transmembrane-spanning domains. The second intracellular loop contains the predicted catalytic ATPase domain comprising four functional domains (A, actuator domain; P1, catalytic phosphorylation site domain; P2 and N, nucleotide binding domains). Locations of disease-associated missense mutations are indicated by stars with homozygous variants in bold. KRS frameshift mutations are indicated by orange and red arrows, with an in-frame exon skipping mutation shown by yellow shading. ***B***, Western blot indicating the buffer solubility of V5-tagged human ATP13A2 transiently expressed in HEK-293T cells. ***C***, Western blot revealing the effect of increasing temperature on the solubility of V5-tagged human ATP13A2 in cell extracts obtained by lysis in 1% Triton X-100 buffer. ***D***, Triton-soluble or Triton-insoluble (RIPA-soluble) fractions derived from HEK-293T cells transiently expressing WT or mutant V5-tagged human ATP13A2 were probed with anti-V5 antibody to monitor the steady-state levels of ATP13A2, or with β-tubulin antibody (or Ponceau S stain) to demonstrate equivalent protein loading. Densitometric analysis indicates dramatically reduced steady-state levels of F182L, G504R and G877R compared with WT ATP13A2 in the Triton-soluble fraction. Bars represent ATP13A2 levels normalized to β-tubulin (mean±SEM; *n* = 3–4 experiments) expressed in arbitrary units. ***E***, Western blot analysis of the detergent-soluble fraction from SH-SY5Y cells transiently expressing WT or mutant V5-tagged ATP13A2. Densitometric analysis of ATP13A2 levels normalized to β-tubulin reveals reduced levels of F182L, G504R and G877R compared with WT ATP13A2 (mean±SEM; *n* = 3 experiments). ***F***, Detergent-soluble extracts from HEK-293T cell transiently expressing WT and KRS mutant V5-tagged human ATP13A2 were probed with anti-V5 antibody, or β-tubulin antibody as a loading control. KRS mutants exhibit markedly reduced steady-state levels and produce truncations due to the loss of three (3057delC) or six (1632_1653dup22) C-terminal transmembrane domains, or the in-frame skipping of exon 13 (1306+5G→A) that removes half of the third transmembrane domain [5]. Molecular weight markers are indicated in kilodaltons (kDa). *P<0.05, **P<0.01 or non-significant (*ns*) compared to WT ATP13A2 by one-way ANOVA with Newman-Keuls post-hoc analysis.

To begin to explore the potential pathogenic effects of missense mutations in ATP13A2 associated with early-onset parkinsonism, we generated expression constructs for V5-tagged human ATP13A2 harboring homozygous (F182L, G504R and G877R) or heterozygous (T12M, G533R and A746T) missense mutations (Figure 1A). We also introduced a synthetic D513N mutation that ablates a critical P1 domain phospho-acceptor site required for ATPase activity that is predicted to be non-functional (Figure 1A) [16], [18]. To initially explore the biochemical properties of ATP13A2, the solubility of these variants in different extraction buffers at ambient temperature was determined by Western blotting from HEK-293T cell extracts transiently expressing V5-tagged ATP13A2. ATP13A2 is most soluble in 1% Triton X-100 buffer and Laemmli sample buffer consistent with being a transmembrane-spanning protein (Figure 1B). We also assessed the effects of temperature on the solubility of ATP13A2 derived from HEK-293T cells. Increasing temperature from 60 to 90°C decreases the solubility of ATP13A2 in 1% Triton X-100 buffer (Figure 1C). Therefore, for all subsequent experiments we routinely employed buffer containing 1% Triton X-100 without heating to efficiently extract ATP13A2 from cells. Frameshift and splicing mutations in ATP13A2 associated with KRS (i.e. 3057delC, 1632_1653dup22 and 1306+5G→A) produce truncated proteins that are unstable and degraded by the proteasome [5], [13]. To explore the effects of missense mutations on protein stability, we examined the steady-state levels of ATP13A2 variants in HEK-293T cells by Western blotting. The homozygous mutations, F182L, G504R and G877R, produce a significant decrease in the steady-state levels of Triton-soluble ATP13A2 compared to WT protein (Figure 1D). The heterozygous T12M, G533R and A746T mutations or the non-functional variant, D513N, do not significantly influence the levels of ATP13A2 protein (Figure 1D). Similar observations were made for the steady-state levels of Triton-insoluble ATP13A2 thereby demonstrating that missense mutations do not influence the detergent solubility of ATP13A2 (Figure 1D). The homozygous mutations, F182L, G504R and G877R, similarly reduce the steady-state levels of ATP13A2 in the Triton-soluble fraction of human SH-SY5Y neural cells (Figure 1E). For comparison, we also assessed the stability of ATP13A2 harboring KRS truncation mutations. The 3057delC, 1632_1653dup22 and 1306+5G→A mutations markedly reduce the steady-state levels of ATP13A2 to levels comparable to the effects of the F182L, G504R and G877R mutations (Figure 1F). To determine the effects of missense mutations on the steady-state mRNA expression levels of human ATP13A2 we conducted quantitative RT-PCR for each ATP13A2 variant expressed in HEK-293T cells. With the exception of the G877R variant, WT and mutant ATP13A2 mRNAs are expressed at similar levels (Figure S1). G877R ATP13A2 exhibits a non-significant reduction in mRNA expression levels suggesting that this mutation could potentially influence mRNA transcription and/or stability, which contributes in part to the markedly reduced steady-state protein levels observed for this variant (Figure 1D and 1E). Collectively, our data demonstrate that homozygous missense mutations associated with early-onset parkinsonism commonly reduce the steady-state levels of ATP13A2 consistent with reduced protein stability.

### Homozygous missense mutations impair the protein stability of ATP13A2

**Figure 2:**
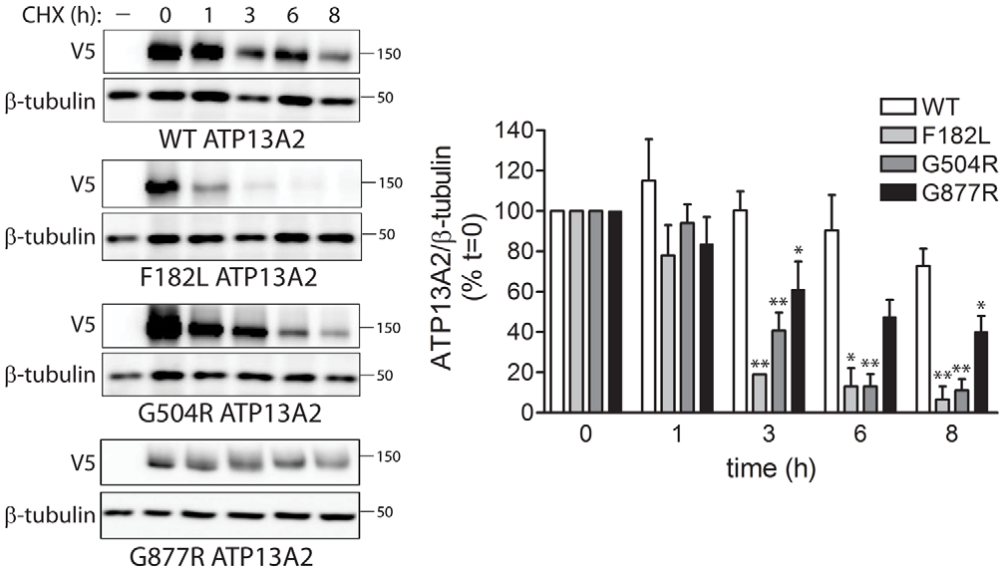
Homozygous missense mutations enhance the protein turnover of ATP13A2. Disease-associated homozygous mutations (F182L, G504R and G877R) enhance the turnover of ATP13A2. HEK-293T cells transiently expressing V5-tagged human ATP13A2 (WT or mutant) were treated with cycloheximide (CHX, 200 µg/ml) at 24 h post-transfection, and cells were harvested at 0, 1, 3, 6 and 8 h thereafter. Equivalent detergent-soluble extracts were probed with anti-V5 antibody to monitor ATP13A2 turnover, or with anti-β-tubulin antibody to demonstrate equal protein loading. Densitometric analysis indicates the markedly increased turnover of F182L, G504R and G877R variants compared to WT ATP13A2. The levels of ATP13A2 were normalized to β-tubulin. Bars represent ATP13A2 levels normalized to β-tubulin (mean±SEM; *n* = 3–4 experiments) expressed as a percent of time point ′0′ for each variant. *P<0.05 or **P<0.01 compared to WT ATP13A2 within each time interval by one-way ANOVA with Newman-Keuls post-hoc analysis.

Since homozygous missense mutations dramatically reduce the steady-state levels of ATP13A2, we next sought to determine whether these effects are due to impaired protein stability. HEK-293T cells transiently expressing human ATP13A2 variants (WT, F182L, G504R and G877R) were treated with cycloheximide (CHX) to inhibit new protein synthesis and sampled at 1, 3, 6 and 8 hours post-treatment to estimate the rate of protein turnover by Western blotting. The levels of WT ATP13A2 are reduced by ∼25% following treatment with CHX for 8 hours (Figure 2). In contrast, the F182L and G504R variants are dramatically reduced by ∼90% after treatment with CHX for 8 hours with an estimated half-life of <3 hours, whereas the G877R variant is modestly reduced by ∼60% over 8 hours (Figure 2). The turnover of the F182L and G504R ATP13A2 variants correlates closely with their reduced steady-state protein levels (refer to Figure 1D and 1E), whereas the reduced steady-state protein levels of the G877R mutant result from a combination of reduced protein stability (Figure 2) and reduced mRNA expression levels (Figure S1). Our data demonstrate that homozygous missense mutations markedly impair the protein stability of ATP13A2 in support of a loss-of-function mechanism of action for these mutations.

### Homozygous missense mutations enhance the proteasomal degradation of ATP13A2

**Figure 3:**
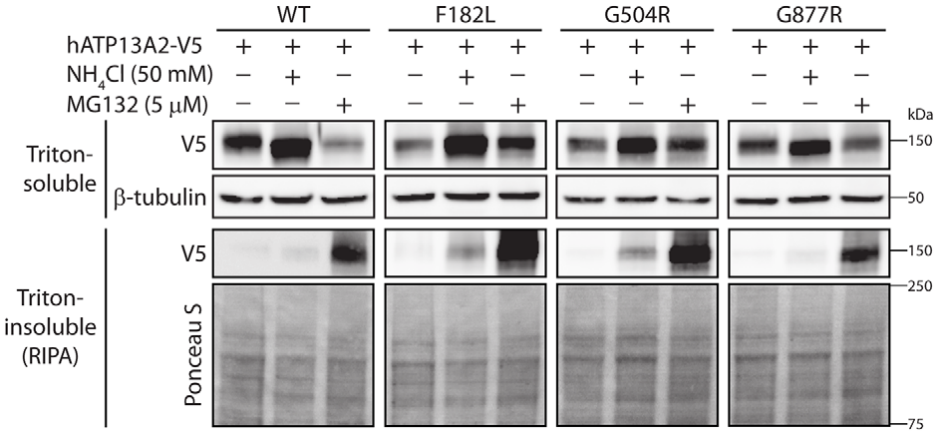
Homozygous missense mutations enhance the degradation of ATP13A2 by the proteasome. HEK-293T cells transiently expressing V5-tagged human ATP13A2 (WT, F182L, G504R and G877R) were treated with the proteasome inhibitor, MG132 (5 µM), or the lysosome inhibitor, ammonium chloride (NH_4_Cl, 50 mM), for 24 h. Triton-soluble and Triton-insoluble (RIPA-soluble) fractions were probed with anti-V5 antibody to monitor ATP13A2 levels, or with β-tubulin antibody (or Ponceau S stain) to demonstrate equivalent protein loading. Inhibition of the proteasome leads to the marked recovery of F182L and G504R protein variants compared to WT and G877R ATP13A2 in the Triton-soluble and -insoluble fractions. Molecular weight markers are indicated in kilodaltons (kDa). Blots are representative of at least four independent experiments.

To understand the mechanism leading to the destabilization of ATP13A2 due to homozygous missense mutations, we assessed the degradation of ATP13A2 variants through the proteasomal and lysosomal pathways. HEK-293T cells transiently expressing human ATP13A2 variants (WT, F182L, G504R and G877R) were treated for 24 hours with the proteasomal inhibitor, MG132 (5 µM) or the lysosomal inhibitor, ammonium chloride (50 mM). The steady-state levels of Triton-soluble and Triton-insoluble ATP13A2 were monitored by Western blotting. The levels of WT, F182L, G504R and G877R ATP13A2 in the Triton-soluble fraction are markedly increased by lysosome inhibition (Figure 3) suggesting that ATP13A2 is normally degraded by the lysosomal pathway. Proteasomal inhibition results in an increase of F182L and G504R ATP13A2 in the Triton-soluble fraction that is more pronounced in the Triton-insoluble fraction compared to the WT and G877R proteins (Figure 3). Collectively, our data suggest that ATP13A2 is normally subjected to lysosomal degradation consistent with the known localization of this protein to lysosomes [5]. Furthermore, the homozygous missense mutations, F182L and G504R, promote the proteasomal degradation of ATP13A2 consistent with their reduced protein stability.

### Localization of ATP13A2 to endosomal and lysosomal vesicles in neurons

**Figure 4:**
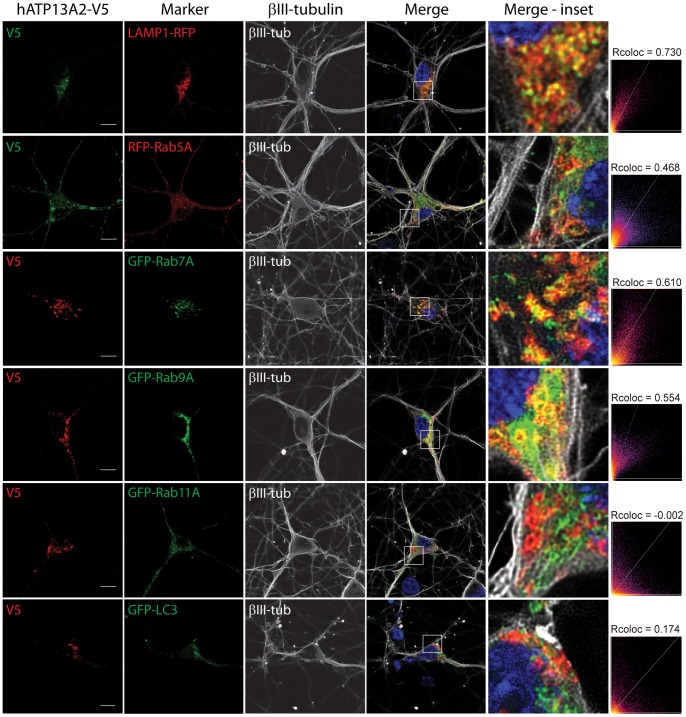
Co-localization of ATP13A2 with endosomal and lysosomal vesicular membranes in neurons. Confocal fluorescence microscopy reveals the co-localization of V5-tagged human ATP13A2 with lysosomal membranes (LAMP1-RFP), early (RFP-Rab5A) and late (GFP-Rab7A and GFP-Rab9A) endosomal compartments but fails to appreciably co-localize with recycling endosomes (GFP-Rab11A) or autophagosomes (GFP-LC3) within the soma of rat primary cortical neurons. Staining for the neuronal marker, βIII-tubulin, and nuclei (DAPI) are also indicated. Cytofluorograms and correlation coefficients (Rcoloc) indicate the extent of co-localization between ATP13A2 and vesicular markers. Confocal images are taken from a single z-plane at 0.15 µm thickness. Images are representative of at least two independent transfection experiments. Scale bar: 10 µm.

ATP13A2 has been shown to localize to lysosomal membranes in mammalian cells [5], [11], [13], [17]. To explore the subcellular localization of ATP13A2 in neurons, we assessed the co-localization of exogenous ATP13A2 with various vesicular markers in rat primary cortical neurons by confocal fluorescence microscopy. Cortical neurons were co-transfected with V5-tagged human ATP13A2 and fluorescent fusion proteins (LAMP1-RFP, RFP-Rab5A, GFP-Rab7A, GFP-Rab9A, GFP-Rab11A and GFP-LC3) at a 10∶1 DNA molar ratio, and subjected to immunocytochemistry. Human ATP13A2 adopts a punctate distribution pattern within the soma and processes of cortical neurons consistent with an intracellular vesicular localization (Figure 4). ATP13A2 co-localizes to a large extent with LAMP1-RFP, a marker of lysosomal membranes, predominantly within the neuronal soma (Figure 4). We also explored the co-localization of ATP13A2 with additional vesicular compartments related to the lysosomal pathway. Human ATP13A2 markedly co-localizes with GFP-Rab7A- and GFP-Rab9A-positive late endosomal compartments and RFP-Rab5A-positive early endosomes but fails to appreciably co-localize with GFP-Rab11A-positive recycling endosomes or GFP-LC3-positive autophagosomes in cortical neurons (Figure 4). Our data demonstrate that ATP13A2 normally co-localizes with intracellular vesicular compartments in neurons, including lysosomes and early and late endosomes.

### Homozygous missense mutations disrupt the vesicular localization of ATP13A2

**Figure 5:**
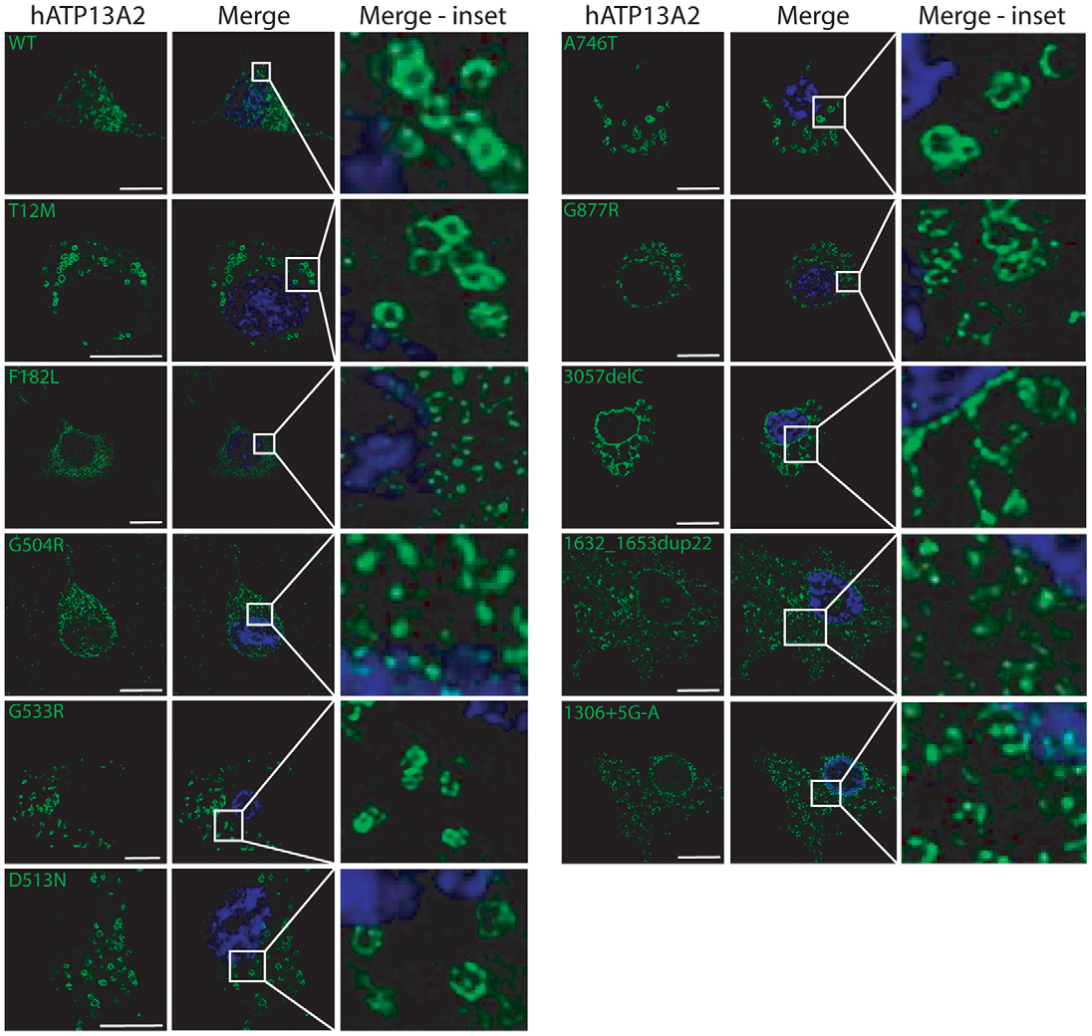
Homozygous missense mutations disrupt the vesicular membrane localization of ATP13A2. Confocal fluorescence microscopy reveals the cytoplasmic vesicular localization of V5-tagged human ATP13A2 variants transiently expressed in rat primary cortical neurons. WT or missense mutant (T12M, D513N, G533R, A746T or G877R) forms of ATP13A2 adopt a similar localization to large vesicular membranes, whereas F182L and G504R missense mutants or KRS mutants (3057delC, 1632_1653dup22 or 1306+5G→A) abnormally localize to small diffuse punctate structures. Staining for ATP13A2 (anti-V5 antibody, green) and nuclei (DAPI, blue) are indicated. Confocal images are taken from a single z-plane at 0.15 µm thickness. Images are representative of at least two independent transfection experiments. Scale bar: 10 µm.

**Figure 6:**
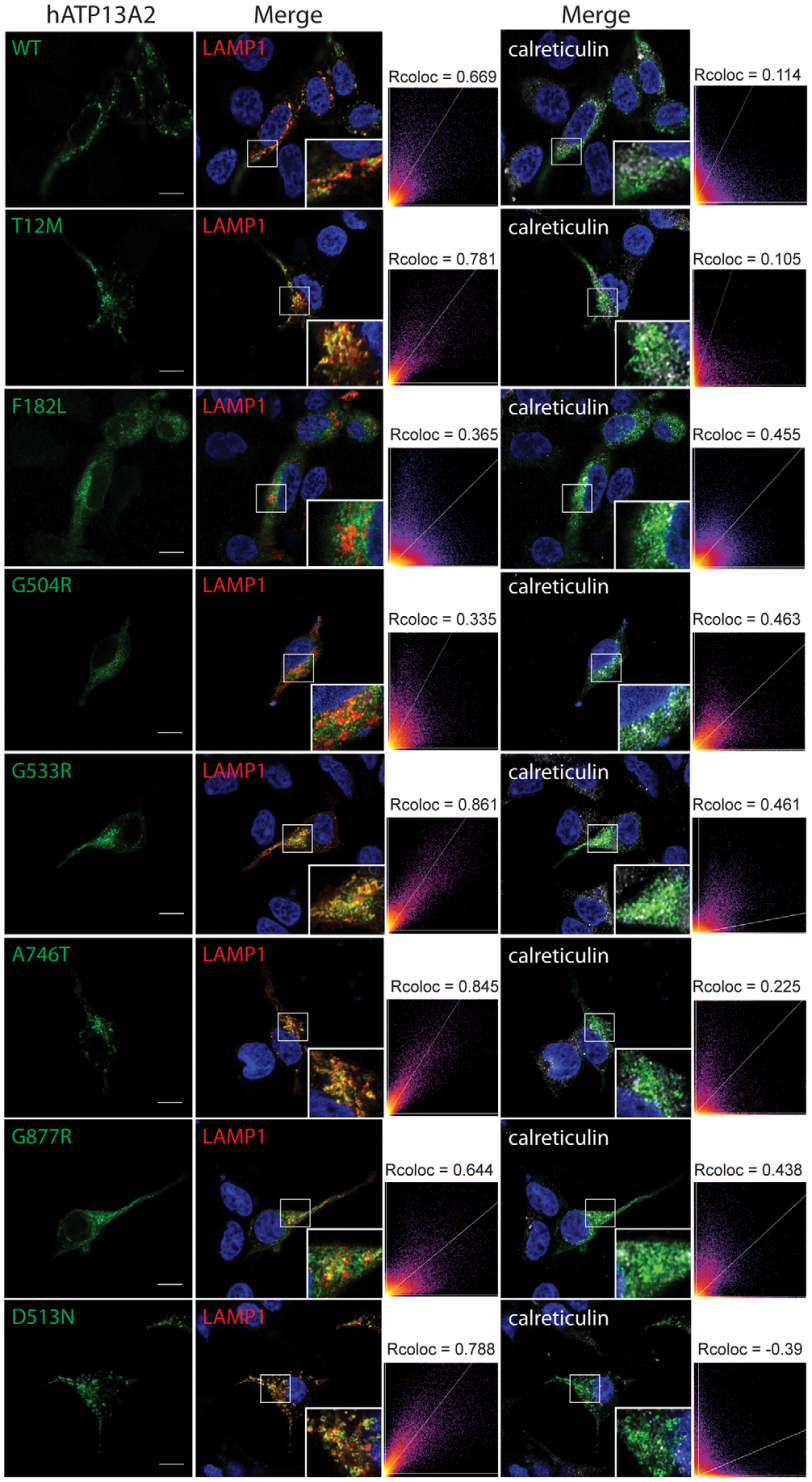
Homozygous F182L and G504R mutations induce the mislocalization of ATP13A2 to the endoplasmic reticulum. Confocal fluorescence microscopy reveals the enhanced co-localization of F182L and G504R mutant ATP13A2 with the endoplasmic reticulum marker, calreticulin, and reduced co-localization with lysosomes (LAMP1-RFP), compared to WT or missense mutant (T12M, D513N, G533R, A746T or G877R) ATP13A2 transiently expressed in human SH-SY5Y neural cells. Staining for V5-tagged human ATP13A2 (V5, green), LAMP1-RFP (red), calreticulin (gray) and nuclei (DAPI, blue) are indicated. Cytofluorograms and correlation coefficients (Rcoloc) indicate the extent of co-localization of ATP13A2 variants with lysosomal (LAMP1) and ER (calreticulin) markers. Confocal images are taken from a single z-plane at 0.15 µm thickness. Images are representative of at least two independent transfection experiments. Scale bar: 10 µm.

KRS mutations which produce truncated forms of ATP13A2 cause the mislocalization of ATP13A2 from intracellular vesicles to the ER where they are degraded by the ERAD pathway [5], [13]. To determine whether disease-associated missense mutations potentially act through a similar mechanism, we explored the subcellular localization of human ATP13A2 variants expressed in rat primary cortical neurons by confocal fluorescence microscopy. WT and mutant (T12M, G533R D513N, A746T and G877R) ATP13A2 adopt a similar distribution to large vesicular membranes throughout the cytoplasm of neuronal soma (Figure 5). In contrast, the F182L and G504R mutants localize to smaller diffuse punctate structures similar to the truncated KRS mutants, 3057delC, 1632_1653dup22 and 1306+5G→A, consistent with mislocalization to the ER (Figure 5). To further explore the mislocalization of ATP13A2 missense mutants, we examined the co-localization of V5-tagged ATP13A2 variants with the exogenous lysosomal marker, LAMP1-RFP, or the endogenous ER marker, calreticulin, in human SH-SY5Y neural cells (Figure 6). WT and mutant (T12M, G533R D513N, A746T and G877R) ATP13A2 markedly co-localize with LAMP1, whereas in comparison, the F182L and G504R mutants reveal markedly reduced co-localization with LAMP1 and increased co-localization with calreticulin thereby demonstrating mislocalization of these mutant proteins at least in part to the ER (Figure 6). Our data reveal that homozygous missense (F182L and G504R) or KRS mutations disrupt the normal localization of ATP13A2 to vesicular membranes in cortical neurons and neural cell lines, whereas additional heterozygous (T12M, G533R or A746T), homozygous (G877R) or non-functional (D513N) mutations do not appreciably influence the vesicular localization of ATP13A2.

### Heterozygous missense mutations impair the ATPase activity of ATP13A2

**Figure 7:**
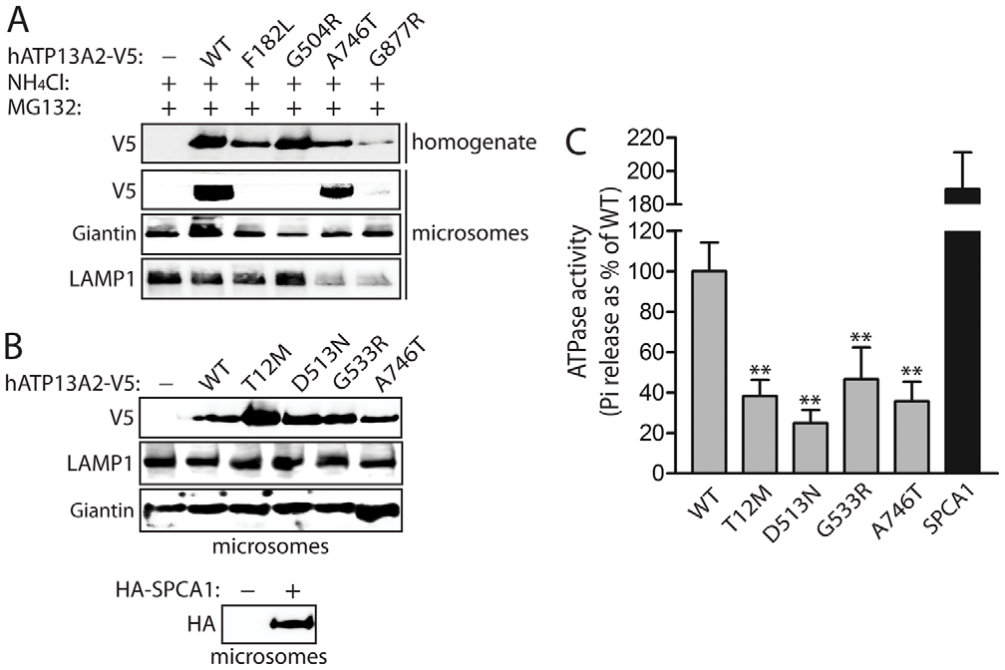
Heterozygous missense mutations impair the ATPase activity of ATP13A2. ***A***, Microsomal fractions prepared by ultracentrifugation from HEK-293T cells transiently expressing V5-tagged human ATP13A2 variants (or empty vector control) following treatment for 24 hours with 5 µM MG132 and 50 mM NH_4_Cl to inhibit proteasomal and lysosomal degradation. Microsomes or total homogenates were probed with anti-V5 antibody to monitor ATP13A2 levels, or with anti-LAMP1 and anti-Giantin antibodies to detect enrichment of lysosomal and Golgi membranes, respectively. Homozygous mutants (F182L, G504R and G877R) fail to localize to microsomes. ***B***, Microsomal fractions from HEK-293T cells expressing WT or mutant V5-tagged ATP13A2 or HA-tagged human SPCA1 were probed with anti-V5 or anti-HA antibodies to monitor each protein variant, or with anti-LAMP1 and anti-Giantin antibodies to detect membranes. ***C***, ATP hydrolysis activity of ATP13A2 variants or SPCA1 was determined in microsomal fractions by measuring the concentration of free phosphate (Pi) released from ATP. The levels of ATP13A2 in each microsomal fraction were assessed by Western blot analysis with anti-V5 antibody, quantified by densitometry, and used for normalization of Pi release between ATP13A2 variants. ATP hydrolysis activity is expressed as Pi release as a percent of WT ATP13A2 activity. Bars represent the mean±SEM (*n* = 4 experiments). **P<0.01 compared to WT ATP13A2 by one-way ANOVA with Newman-Keuls post-hoc analysis.

The majority of missense mutations in ATP13A2 are located within the second large intracellular loop that contains the ATPase enzymatic domain (refer to Figure 1A). To explore the effects of missense mutations on ATPase activity, we first prepared microsomal fractions containing vesicular membranes including lysosomes from HEK-293T cells transiently expressing human ATP13A2 variants, since we anticipate that ATP13A2 would be fully active within vesicular membranes. Compared to WT ATP13A2, the F182L, G504R and G877R mutants are not appreciably detected in microsomes even following combined proteasome and lysosome inhibition to prevent their degradation (Figure 7A). Heterozygous mutations (T12M, G533R and A746T) or the ATPase-deficient D513N mutant were normally enriched in microsomal fractions together with markers of lysosomal (LAMP1) and Golgi (giantin) membranes (Figure 7B). As a positive control, we employed human Secretory Pathway Ca^2+^-ATPase 1 (SPCA1/ATP2C1), a related P-type ATPase localized to Golgi membranes [20], [21], which could also be detected in microsomes (Figure 7B). To assess ATPase activity, microsomal fractions were used for *in vitro* assays to monitor ATP13A2-mediated ATP hydrolysis by measuring the release of free γ-phosphate produced by hydrolysis of ATP to ADP. ATPase assays were conducted in the presence of bafilomycin A1 (5 µM) to specifically inhibit the activity of the major vacuolar-type H^+^-ATPase (V-ATPase) that is present in vesicular membranes [22]. Microsomes expressing WT ATP13A2 markedly enhance ATP hydrolysis compared to control microsomes, whereas microsomes containing SPCA1 exhibit substantially increased ATPase activity (Figure 7C), as previously described [23]. The D513N mutant dramatically impairs the ATP hydrolysis activity of ATP13A2 (Figure 7C), consistent with disruption of the phospho-acceptor site in the P1 domain that is critically required for ATPase activity [16], [18]. Importantly, microsomes expressing the heterozygous T12M, G533R or A746T mutants display significantly reduced ATPase activity compared to WT ATP13A2 (Figure 7C). Our data suggest that heterozygous missense mutations impair the ATPase activity of ATP13A2.

### Missense mutations impair the effects of ATP13A2 on neurite outgrowth

**Figure 8:**
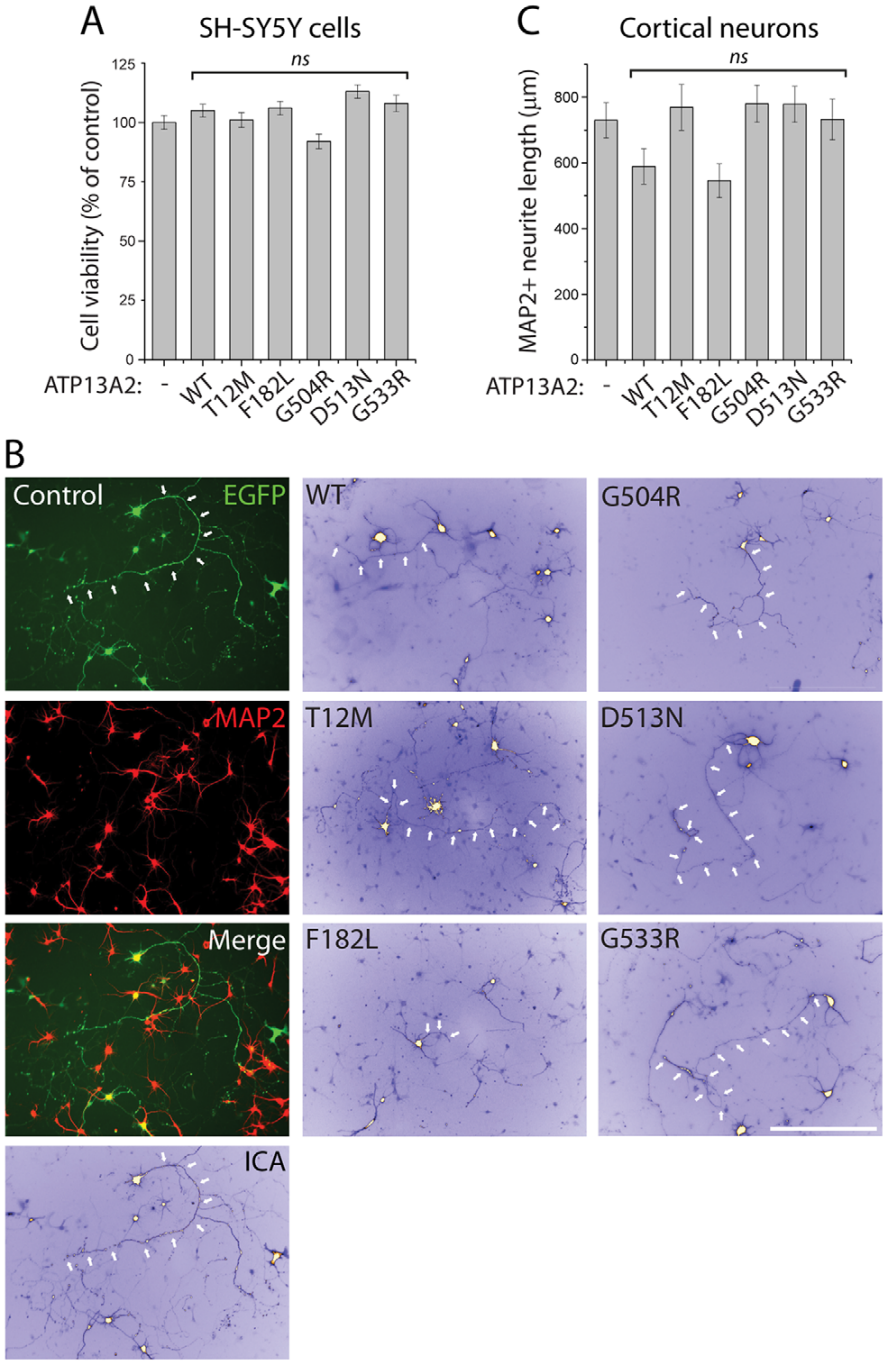
Overexpression of ATP13A2 impairs neurite outgrowth of cortical neurons. *A* , Viability of SH-SY5Y cells transiently expressing V5-tagged human ATP13A2 variants at 48 h post-transfection assessed by MTS assay. Bars represent mean±SEM (*n* = 4 experiments). ***B***, Rat primary cortical neurons co-transfected at DIV 3 with V5-tagged human ATP13A2 variants (or control empty vector) and EGFP plasmids at a DNA molar ratio of 10∶1. Cultures were fixed at DIV 6. Representative fluorescent micrographs reveal the co-labeling of cortical neurons with EGFP and MAP2 for each condition. EGFP images representing ATP13A2-positive cortical neurons were pseudo-colored with ICA to improve the contrast of neuritic processes for length measurements. Axonal processes are indicated with arrows. Scale bar: 400 µm. ***C***, Analysis of the length of EGFP+/MAP2+ neurites from rat primary cortical neurons expressing V5-tagged human ATP13A2 variants (or control empty vector). Expression of WT or F182L ATP13A2 produces a non-significant shortening of cortical axonal processes compared to control neurons expressing EGFP alone. Bars represent the length of MAP2+ neurites in µm (mean±SEM; *n* = 67–88 neurons from three independent cultures). Non-significant (*ns*) compared to control (-) by one-way ANOVA with Newman-Keuls post-hoc analysis.

KRS mutations in ATP13A2 have been shown to induce cellular toxicity, ER stress and sensitize cells to ER stress-induced cell death [11], [13]. To initially assess the effects of ATP13A2 missense mutants on cell viability, we examined the effects of overexpressing human ATP13A2 variants on the viability of human SH-SY5Y neural cells using an MTS proliferation assay. The expression of WT or missense mutant forms of ATP13A2 does not appreciably influence the basal viability of SH-SY5Y cells (Figure 8A) demonstrating that ATP13A2 missense mutants are not intrinsically toxic to mammalian cells unlike truncated KRS mutants. We next sought to compare the subtle effects of ATP13A2 variants on neuronal integrity by assessing neurite outgrowth. Rat primary cortical cultures at DIV 3 were transiently co-transfected with V5-tagged human ATP13A2 variants (or empty vector) and EGFP at a 10∶1 molar ratio to label transfected neurons. After 72 hours, cultures were fixed and processed for immunocytochemistry to identify MAP2-positive cortical neurons. For each ATP13A2 variant, the length of EGFP+/MAP2+ cortical neurites was determined (Figure 8B). The overexpression of WT and F182L mutant ATP13A2 leads to a non-significant reduction of cortical neurite length compared to control MAP2+ neurons expressing EGFP alone, whereas expression of the T12M, G504R, D513N and G533R variants do not differ from EGFP alone (Figure 8C). Western blot analysis confirmed that the levels of each human ATP13A2 variant in cortical neurons were similar to those achieved in HEK-293T cells (data not shown). Taken together, our data demonstrate that the overexpression of WT ATP13A2 displays a non-significant trend towards impairing neurite outgrowth whereas missense mutations in ATP13A2, with the exception of the F182L variant, disrupt this neurite effect consistent with a loss-of-function mechanism of action. Our data further suggest that the F182L variant may act through both a loss-of-function (i.e. impaired protein stability) and a gain-of-function (i.e. impaired neurite outgrowth) mechanism similar to KRS truncation mutations [11], [13].

### ATP13A2 overexpression sensitizes cortical neurons to neurite shortening induced by heavy metals

**Figure 9:**
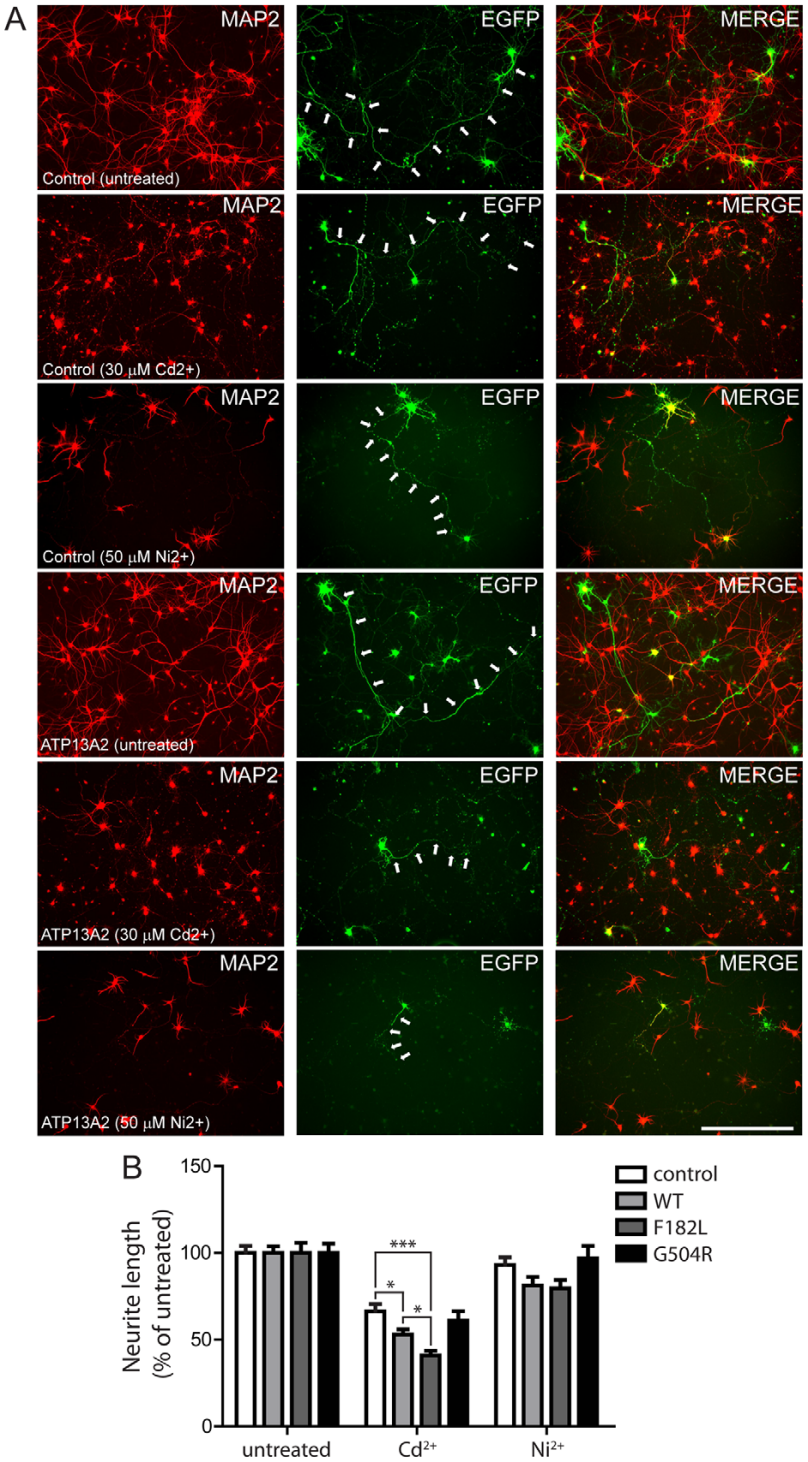
Overexpression of ATP13A2 sensitizes cortical neurons to heavy metal-induced neurite shortening. *A* , Rat primary cortical neurons were co-transfected at DIV 3 with V5-tagged human ATP13A2 variants (or control empty vector) and EGFP plasmids at a DNA molar ratio of 10∶1. Cultures were treated without or with cadmium (Cd^2+^, 30 µM) or nickel (Ni^2+^, 50 µM) for 24 h and then fixed at DIV 6. Representative fluorescent micrographs reveal the co-labeling of MAP2+ cortical neurons with EGFP for each condition. Axonal processes are indicated with arrows. Scale bar: 400 µm. ***B***, Analysis of the length of EGFP+/MAP2+ neurites reveals that the overexpression of WT or F182L ATP13A2 sensitizes cortical neurons to neurite shortening induced by treatment with 30 µM Cd^2+^ or 50µM Ni^2+^ for 24 h, compared to neurons expressing G504R ATP13A2 or EGFP alone (control). Data represent neurite length (mean±SEM; control, *n* = 73–156 neurons; cadmium, *n* = 39–119 neurons; nickel, *n* = 32–72 neurons; from three independent cultures) expressed as a % of untreated neurons (control or ATP13A2 overexpression). *P<0.05 or ***P<0.001 as indicated by one-way ANOVA with Newman-Keuls post-hoc analysis.

Since the overexpression of human ATP13A2 tends to impair the neurite outgrowth of primary cortical neurons, we next assessed the impact of exposure to heavy metals on this neuronal phenotype to assess the potential functional interaction between ATP13A2 and metal cations. Primary cortical neurons were co-transfected with V5-tagged human ATP13A2 variants (or empty vector) and EGFP plasmids at a 10∶1 molar ratio, treated after 48 hours with or without Cd^2+^ (30 µM) or Ni^2+^ (50 µM) for 24 hours, and subjected to immunocytochemistry to label MAP-positive neurons. As before, we determined the length of EGFP+/MAP2+ neurites for each condition (Figure 9A). In control neurons expressing EGFP alone, treatment with 30 µM Cd^2+^ results in a ∼33% reduction of neurite length compared to untreated neurons (Figure 9B). In contrast, Cd^2+^ treatment produces a marked ∼47% reduction of neurite length in neurons expressing WT ATP13A2, a ∼59% reduction with the F182L mutant, and a ∼39% reduction with the G504R mutant (Figure 9B). Treatment with 50 µM Ni^2+^ induces a ∼7% reduction of neurite length of control neurons expressing EGFP alone, whereas neurons expressing WT, F182L or G504R ATP13A2 exhibit a ∼19%, ∼20% or ∼3% reduction of neurite length, respectively, compared to untreated neurons (Figure 9B). Mn^2+^ treatment fails to induce neurite shortening in the presence or absence of ATP13A2 and was not studied further (data not shown). Collectively, our data demonstrate that the overexpression of ATP13A2 sensitizes neurons to impairments of neurite outgrowth induced by exposure to cadmium or nickel ions. The F182L mutation enhances the effects of cadmium compared to WT ATP13A2 further suggesting enhanced neuronal toxicity of this mutant protein, whereas the G504R mutation acts in a loss-of-function manner. These data support a functional interaction of ATP13A2 with heavy metal ions in post-mitotic neurons.

## Discussion

Homozygous and heterozygous missense mutations in ATP13A2 are associated with early-onset forms of parkinsonism [9], [10], [14], [15]. The pathogenic mechanism(s) by which these mutations may cause disease have not been elucidated. For heterozygous mutations, it unclear whether they represent rare benign polymorphic variants or authentic disease-causing variants. Here, we demonstrate that homozygous missense mutations (F182L and G504R) commonly exhibit reduced steady-state levels, increased turnover, and enhanced proteasomal degradation of ATP13A2 consistent with impaired protein stability. The homozygous G877R mutation exhibits a combination of reduced protein stability and reduced mRNA expression levels. In contrast, heterozygous mutations, T12M, G533R and A746T, have small or minimal effects on protein stability. We further demonstrate that ATP13A2 is normally localized to cytoplasmic acidic vesicular membranes in mammalian neurons, including lysosomes and early and late endosomes. Similar to truncating KRS mutations [5], [11], the highly unstable F182L and G504R mutations disrupt the cytoplasmic vesicular localization of ATP13A2 and promote its mislocalization to the ER. Heterozygous mutations (T12M, G533R and A746T) impair the ATPase activity of microsome-localized ATP13A2 whereas the homozygous F182L, G504R and G877R mutations fail to appreciably localize within microsomes. In contrast to KRS mutations in ATP13A2 [11], [13], missense mutants are not intrinsically toxic to human cell lines. However, in primary cortical neurons, the overexpression of wild-type ATP13A2 compromises neuronal integrity by impairing neurite outgrowth whereas missense mutations (with the exception of F182L) lack this toxic effect consistent with a loss-of-function mechanism for these mutations. ATP13A2 overexpression further sensitizes neurons to neurite shortening induced by exposure to the heavy metal ions, cadmium and nickel, with the F182L or G504R mutations enhancing or reducing this sensitizing effect, respectively. Collectively, our study reveals differential pathogenic effects of missense mutations on ATP13A2 protein stability, subcellular localization, ATPase activity and neuronal phenotypes consistent with a common loss-of-function mechanism for the actions of homozygous and heterozygous mutations.

Homozygous missense mutations (i.e. F182L and G504R) markedly impair the protein stability of ATP13A2. Similar to truncating KRS mutations (i.e. 1632_1653dup22, 3057delC, 1306+5G→A and 3253delC) that mislocalize to the ER [5], [13], the F182L and G504R mutations impair the normal localization of ATP13A2 to vesicular membranes and facilitate mislocalization to the ER suggesting that these mutant proteins are potentially misfolded and may be subjected to degradation by the ERAD pathway. The G877R mutation, although partly unstable, can normally localize to vesicular membranes suggesting that it may not be a major substrate of the ERAD pathway. The F182L and G504R mutations appear to enhance the turnover of ATP13A2 by promoting its proteasomal degradation. The F182L, G504R and G877R mutations are localized to the first extracellular (F182L) and second intracellular loop (G504R and G877R) regions of ATP13A2 and most likely cause conformational alterations to these loops that destabilize the protein. Two of these mutations result in the non-conservative substitution of a neutral glycine residue for a positively charged arginine residue perhaps suggesting that simply altering the charge of this loop or increasing the side chain length has a destabilizing conformational effect that varies in magnitude depending on the location of this residue within the loop i.e. G504R>G877R. The effect of substituting a hydrophobic phenylalanine residue for a similarly hydrophobic leucine residue at position 182 is less obvious but may relate to the reduced bulk of the side chain due to the absence of an aromatic benzene ring. Potentially, disease-associated missense mutations may produce a non-functional protein that is selectively targeted for degradation. Of note, the G504R variant is located adjacent to the P1 domain whereas the G877R variant occupies the P2 domain, with both domains implicated to play a role in the ATPase activity of ATP13A2 and may therefore produce a non-functional protein. Three heterozygous mutations localized to the intracellular N-terminal tail (T12M) and the intracellular catalytic loop (G533R and A746T) fail to have obvious effects on protein stability or subcellular localization suggesting that they may act through an alternate mechanism. Accordingly, heterozygous mutations (T12M, G533R and A746T) commonly impair the ATPase activity of microsomal-localized ATP13A2 whereas the homozygous F182L, G504R and G877R mutants fail to appreciably localize to microsomes and were thus refractory to analysis of ATPase activity. Missense mutations therefore appear to commonly act through a loss-of-function mechanism by exerting differential effects on the protein stability, subcellular localization or ATPase activity of ATP13A2.

A potential caveat of the present study is that it relies on the transient overexpression of human ATP13A2 variants in human cell lines and cultured primary neurons at non-physiological expression levels. Consistent with our study, recent studies of KRS truncation mutants using similar overexpression approaches in a range of mammalian cells have revealed a destabilizing effect on the ATP13A2 protein resulting from its mislocalization to the ER and enhanced proteasomal degradation [5], [11], [13], [24]. Collectively, there is strong agreement that PD-associated missense mutations and KRS mutations act through similar mechanisms to produce a loss-of-function effect. It will be important to confirm the destabilizing effects of missense mutations and truncating KRS mutations on the ATP13A2 protein in physiologically-relevant systems such as patient-derived cells or tissues, or knockin mouse models. Along these lines, cultured olfactory neurospheres harboring compound heterozygous ATP13A2 mutations (i.e. 3253delC frameshift and L1059R missense mutations) reveal a reduction in endogenous ATP13A2 mRNA levels primarily due to nonsense-mediated decay of the 3523delC mutant mRNA although ATP13A2 protein levels were not assessed in this model [11]. The impact of disease-associated mutations on the stability of the endogenous ATP13A2 protein awaits confirmation in future studies pending the availability of appropriate cellular models or tissues. PD-associated and KRS mutations share similar biochemical effects suggesting that ATP13A2 loss-of-function is primarily responsible for precipitating neurodegeneration. Recent studies have employed ATP13A2 knockdown in cultured cell lines or primary neurons, or patient-derived fibroblasts bearing *ATP13A2* mutations, to model the cellular consequences of disease-associated *ATP13A2* mutations [17], [25], [26], [27]. ATP13A2 knockdown has been suggested to impair neuronal integrity and/or induce neuronal cell death through a number of potential mechanisms, including abnormal cation homeostasis [17], decreased macroautophagy [26], lysosomal dysfunction [27], α-synuclein accumulation [27], and/or alterations in mitochondrial quality control [17], [25], [26]. Which of these cellular abnormalities are directly responsible for mediating neuronal damage is not yet clear but they most likely result as a consequence of impaired lysosomal activity. Therefore, lysosomal dysfunction potentially underlies the pathogenic effects of ATP13A2 loss-of-function in neurons.

We find that the overexpression of ATP13A2 missense mutants in human SH-SY5Y neural cells does not influence cell viability suggesting that they are not intrinsically toxic. KRS mutants have been shown to induce cell death in HeLa cells where they also sensitize to ER stress-induced cell death [13]. However, another study reports the lack of cell death induced by the same KRS mutants overexpressed in rat hippocampal neurons [24]. Furthermore, in the context of Mn^2+^-induced cell death in cell lines and hippocampal neurons, WT ATP13A2 protects from cell death whereas KRS mutants lack this ability [24]. Additional toxicity induced by KRS mutants may not therefore play a major role in disease and could relate to overexpression at non-physiological levels, especially given that such mutations lead to a dramatic destabilization and degradation of ATP13A2 [5], [13]. It will be important to determine whether the expression of KRS mutants at physiological levels in patient-derived cells and tissues similarly induces cellular toxicity. A recent study has shown that human olfactory neurospheres harboring compound heterozygous mutations in ATP13A2 (3253delC and L1059R) exhibit signs of ER stress although neuronal viability was not assessed [11]. In primary cortical neurons, we find that the overexpression of WT ATP13A2 tends to impair neurite outgrowth. Consistent with a loss-of-function effect for missense mutations, the disease-associated T12M, G504R and G533R variants fail to influence neurite outgrowth compared to WT ATP13A2. These effects were similar to the effects of an ATPase-deficient D513N variant further supporting a loss-of-function mechanism. Similar to certain unstable KRS truncation mutants [13], the F182L variant appears to induce additional toxicity when overexpressed since it causes a trend of reduced neurite outgrowth of cortical neurons similar to WT ATP13A2 despite its markedly impaired protein stability. It is likely that these effects are exacerbated by overexpression at non-physiological levels in neurons with the primary pathogenic effect of the F182L mutation at physiological expression levels being a loss-of-function due to reduced protein stability. Of note, the heterozygous T12M and G533R variants which have no obvious effects on protein stability or subcellular localization, have no effect on neurite outgrowth suggesting that they may also act as a pathogenic loss-of-function mutations albeit through an alternative mechanism such as via reduced ATPase activity.

In support of a role for ATP13A2 in regulating the transport of Cd^2+^ and Ni^2+^, we find that the overexpression of WT ATP13A2 sensitizes cortical neurons to neurite shortening induced by exposure to these heavy metal ions. This finding was somewhat unexpected as we would anticipate that ATP13A2 might protect against Cd^2+^ and Ni^2+^-induced neuronal toxicity similar to the reported neuroprotective effects of ATP13A2 against Mn^2+^ and α-synuclein toxicity [18], [24]. Consistent with a loss-of-function effect for the G504R mutation, the overexpression of this mutant exhibited a reduced sensitizing effect on neurite length following Cd^2+^ and Ni^2+^ exposure compared to WT ATP13A2. In contrast, the F182L mutation enhanced the sensitizing effect of ATP13A2 following Cd^2+^ exposure consistent with the enhanced neuronal toxicity of this unstable protein following its overexpression. The significance of neurite shortening induced by ATP13A2 is unclear at present and could indicate impaired neuronal integrity due to toxicity or a physiological effect of ATP13A2 in regulating neuronal process morphology similar to the PD-associated protein LRRK2 [28]. Nevertheless, our data support a specific functional interaction of human ATP13A2 with Cd^2+^ and Ni^2+^ suggesting a potential role in the transport of these metal cations in post-mitotic mammalian neurons. Previous studies demonstrate that deletion of the ATP13A2 ortholog, *ykp9*, impairs yeast cell growth in the presence of Cd^2+^, Ni^2+^, Mn^2+^ and Se^2+^ [18], [19]. Furthermore, the expression of WT but not KRS mutant ATP13A2 in mammalian cell lines or rat hippocampal neurons may protect from Mn^2+^-induced apoptotic cell death [24]. Our recent studies have shown that the overexpression of human ATP13A2 in primary cortical neurons delays mitochondrial fragmentation induced by acute exposure to cadmium [17]. Collectively, these studies support a functional interaction between ATP13A2 and heavy metal cations, particularly cadmium. Our future studies will continue to clarify whether ATP13A2 can directly transport heavy metal cations across vesicular membranes, the substrate selectivity of ATP13A2, and the functional significance of cation transport [17].

Collectively, the present study demonstrates that disease-associated homozygous and heterozygous missense mutations in ATP13A2 exhibit differential pathogenic effects on protein stability, subcellular localization, ATPase activity and neuronal phenotypes, thereby providing support for a pathogenic loss-of-function mechanism for these mutations in precipitating early-onset parkinsonism.

## Materials and Methods

### Animals

All animal experiments were approved by the SCAV (Service de la consommation et des affaires veterinaires) in the Canton de Vaud (Animal authorization No. 2293), and conducted in strict accordance with the European Union directive (2010/63/EU) for the care and use of laboratory animals. Animals were maintained in a pathogen-free barrier facility and exposed to a 12 h light/dark cycle with food and water provided *ad libitum*. Pregnant female Sprague-Dawley rats were obtained from Charles River Laboratories (L'Arbresle Cedex, France) and resulting P0 rats were used for preparation of post-natal primary cortical cultures.

### Expression plasmids and antibodies

Mammalian expression plasmids containing full-length V5-tagged human ATP13A2 (WT, 1306+5G→A, 1632_1653dup22 and 3057delC) were kindly provided by Dr. Christian Kubisch (University of Cologne, Germany) [5]. Disease-associated or functional missense mutations (T12M, F182L, G504R, D513N, G533R, A467T and G788R) were introduced into V5-tagged WT ATP13A2 by site-directed mutagenesis using the Stratagene QuickChange II XL kit (Agilent Technologies, La Jolla, CA, USA) according to manufacturer's instructions. The integrity of all constructs was confirmed by DNA sequence analysis. As plasmid controls, a pEGFP-N1 plasmid was obtained from Clontech (Mountain View, CA, USA) and a pcDNA3.1 plasmid was obtained from Invitrogen (Basel, Switzerland). A plasmid expressing HA-tagged human SPCA1 was kindly provided by Dr. Adam Linstedt (Carnegie Mellon University, Pittsburgh, PA) [29]. Expression plasmids containing human EGFP-LC3B (plasmid #11546; [30]), rat LAMP1-RFP (plasmid #1817, [31]), human RFP-Rab5A (plasmid #14437, [32]), human GFP-Rab7A (plasmid #12605; [33]), human GFP-Rab9A (plasmid #12663; [33]) and human GFP-Rab11A (plasmid #12674; [33]) were obtained from Addgene. The following antibodies were employed: mouse monoclonal anti-V5 and anti-V5-peroxidase (Invitrogen); mouse monoclonal anti-MAP2 (clone HM-2) and anti-β-tubulin (clone TUB 2.1), and rabbit polyclonal anti-βIII-tubulin (Sigma-Aldrich, Buchs, Switzerland); mouse monoclonal anti-HA-peroxidase (Roche Applied Science, Basel, Switzerland); mouse monoclonal anti-LAMP1 (clone LY1C6) and rabbit monoclonal anti-calreticulin (clone EPR3924) (EMD Millipore, Billerica, MA, USA); rabbit polyclonal anti-Giantin (ab24586; Abcam, Cambridge, UK); peroxidase-coupled anti-mouse and anti-rabbit IgG, light chain-specific secondary antibodies (Jackson ImmunoResearch, Inc., West Grove, PA, USA); anti-rabbit IgG and anti-mouse IgG coupled to AlexaFluor-488, -546 and -633 (Invitrogen).

### Cell culture and transient transfection

Human SH-SY5Y neuroblastoma cells (CRL-2266; ATCC, Manassas, VA, USA [34], [35]) or HEK-293T cells (Invitrogen) were maintained in Dulbeccòs modified Eagle's Medium (Invitrogen) supplemented with 10% foetal bovine serum (FBS) and 1X penicillin/streptomycin at 37°C in a 5% CO_2_ atmosphere. Cells were transfected with plasmid DNAs using FuGENE HD reagent (Roche Applied Science) according to manufacturer's recommendations. For biochemical assays cells were routinely harvested at 48–72 h post-transfection.

### Primary neuronal cultures

Primary cortical neurons were prepared from rats as previously described [17], [36]. Whole brains were dissected from Sprague-Dawley P0 rats and the cerebral cortices were isolated stereoscopically and dissociated by digestion in media containing papain (20U/ml, Sigma-Aldrich) and mechanical trituration. Cells were plated in 35 mm dishes on glass coverslips coated with poly-*D*-lysine (20 ng/ml; BD Biosciences, Allschwil, Switzerland) and mouse laminin (33 µg/ml; Invitrogen) and cultured in Neurobasal media containing B27 supplement (2% w/v), L-glutamine (500 µM) and penicillin/streptomycin (100 U/ml) (Invitrogen). At *days-in-vitro* (DIV) 3, cortical cultures were treated with cytosine β-*D*-arabinofuranoside (AraC, 10 µM) to inhibit glial cell division. Primary cortical neurons were transfected with plasmid DNAs using Lipofectamine 2000 reagent (Invitrogen) according to manufacturer's instructions to a maximum of 5 µg total DNA per 35 mm dish of cells.

### Cell fractionation and western blotting

For western blotting, HEK-293T or SH-SY5Y cells maintained in growth medium in 6 well plates (250,000 cells/well) were transiently transfected with 2 μg of each plasmid DNA. After 48 h, cells were harvested in media, resuspended in lysis buffer (1X PBS pH 7.4, 1% Triton-X-100, Complete Mini protease inhibitor cocktail [Roche Applied Sciences]) and rotated at 4°C for 1 h. Lysates were centrifuged at 17,500 *g* for 10 min at 4°C, and the resulting pellet and supernatant (Triton-soluble) fractions were collected. The pellet was further solubilized by sonication in RIPA buffer (10 mM Tris-HCl pH 7.4, 150 mM NaCl, 5 mM EDTA, 1% Triton-X-100, 2.5% sodium deoxycholate, 1% SDS, Complete Mini protease inhibitor cocktail [Roche Applied Sciences]) to produce the Triton-insoluble fraction (RIPA-soluble). Triton-soluble and Triton-insoluble fractions were combined 1∶1 with 2X Laemmli sample buffer (Bio-Rad AG, Reinach, Switzerland) containing 5% 2-mercaptoethanol and resolved by SDS-PAGE (7.5%), transferred to Protran nitrocellulose (0.2 μm; Perkin Elmer, Schwerzenbach, Switzerland) and subjected to Western blot analysis. Nitrocellulose membranes were probed with mouse monoclonal anti-V5-peroxidase antibody (Invitrogen), or with mouse monoclonal anti-β-tubulin (Sigma-Aldrich) and peroxidase-coupled anti-mouse IgG (Jackson ImmunoResearch) antibodies to control for protein loading. Ponceau S stain was used to assess protein loading of the Triton-insoluble fraction. Proteins were visualized by enhanced chemiluminescence (ECL; GE Healthcare, Glattbrugg, Switzerland) on a FujiFilm LAS-4000 Luminescent Image Analysis system. Quantitation of protein levels by densitometry was conducted on acquired non-saturated images using LabImage 1D software (Kapelan Bio-Imaging Solutions, Leipzig, Germany).

Where indicated, HEK-293T cells were treated with 5 µM MG132 (Enzo Life Sciences AG, Lausen, Switzerland) or 50 mM ammonium chloride (AppliChem GmbH, Darmstadt, Germany) for 24 h prior to harvesting. For cycloheximide (CHX) assays, CHX (200 μg/ml; Sigma-Aldrich) was added to transfected cells at 24 h post-transfection, and cells were harvested at 0, 1, 3, 6 and 8 h post-treatment.

### Quantitative RT-PCR

Total RNA was purified from HEK-293T cells transiently expressing human ATP13A2 variants using the RNeasy Plus kit (Qiagen, Valencia, CA, USA) and 200 ng of total RNA was subjected to cDNA synthesis using the High Capacity cDNA RT kit (Applied Biosystems, Carlsbad, CA, USA). Quantitative real-time PCR was performed on a 7900HT Real-Time PCR System with SDS 2.3 software (Applied Biosystems) using Power SYBR Green PCR Mastermix (Applied Biosystems). Relative expression was calculated by normalization of plasmid-derived human ATP13A2 expression to β-actin expression. RT minus control samples run in parallel demonstrated negligible contamination from ATP13A2 plasmid DNA. All Q-PCR reactions were performed in triplicate. Primer sequences were as follows: human β-actin, forward 5′-ACCGCGAGAAGATGACCCAGA-3′ and reverse 5′CAGGGATAGCACAGCCTGGATAGCA-3′; plasmid-derived human ATP13A2, forward 5′-GCAGATATCCAGCACAGTGG-3′ and reverse 5′-AGACCGAGGAGAGGGTTAGG-3′.

### ATP hydrolysis assay

ATP hydrolysis activity was measured on microsomal fractions by monitoring the release of free γ-phosphate (Pi) from ATP. Microsomal fractions were prepared from HEK-293T cells transiently expressing V5-tagged ATP13A2 variants or HA-tagged SPCA1 as previously described [23]. Briefly, HEK-293T cells in 6-well plates were harvested in 600 µl hypotonic buffer (10 mM Tris-HCl pH 7.5, 0.5 mM MgCl_2_, 1 mM EDTA, 1X Complete protease inhibitor cocktail [Roche Applied Science]) and kept on ice for 10 min. Cells were homogenized by 40 strokes in a glass Dounce homogenizer, followed by addition of 600 µl solution M (10 mM Tris-HCl pH 7.5, 0.5 M Sucrose, 0.3 M KCl, 6 mM β-mercaptoethanol, 40 µM CaCl_2_) and a further 20 Dounce strokes. Lysates were centrifuged at 8,000 *g* for 20 min at 4°C and the resulting supernatant fraction was centrifuged at 100,000 *g* for 45 min. The resulting microsomal pellet was resuspended in 100 µl solution M1 (10 mM Tris-HCl pH 7.5, 0.25 M Sucrose, 0.15 M KCl, 3 mM β-mercaptoethanol, 20 µM CaCl_2_). Microsomal fractions were quantified by BCA assay (Pierce Biotechnology, Rockford, IL) and 4 µg of microsomal proteins were used for each ATPase assay. ATP hydrolysis activity was measured by monitoring the release of free γ-phosphate (Pi) from ATP in 96-well plates using a high-sensitivity colorimetric ATPase Assay kit (Innova Biosciences, Cambridge, UK) as per manufacturer's recommendations. Activity of the V-ATPase was inhibited by addition of 5 μM Bafilomycin A1 (Sigma-Aldrich) to each microsomal fraction during the reaction. In parallel, replicate microsomal samples without ATP were used to determine non-specific background absorbance. Microsomal fractions from non-transfected cells were used to determine non-specific ATPase activity. Assay samples were incubated with 0.5 mM ATP for 30 min at room temperature, reactions were terminated, and absorbance at 650 nm was measured. Absorbance from replicate samples without ATP was removed from the equivalent sample containing ATP, and the absorbance from non-transfected microsomes was also subtracted from each sample. Microsomal samples were subjected to Western blotting with anti-V5 antibodies and ATP13A2-V5 protein levels were determined by densitometry and used to normalize ATPase activity in each sample. Data represent Pi release from ATP expressed as a percent of WT ATP13A2 activity.

### Immunocytochemistry and confocal microscopy

Immunocytochemical staining was conducted as previously described [36]. Briefly, cells grown on glass coverslips were fixed with 4% paraformaldehyde (PFA) and incubated with primary antibodies for 1–2 h and anti-rabbit or mouse IgG-AlexaFluor-488, -546 or -633 secondary antibodies (Invitrogen) for 1 h. Nuclei were stained with 4′,6-diamidino-2-phenylindole (DAPI; Vector Laboratories, Peterborough, UK). Coverslips were mounted with Mowiol 4–88 mounting medium (Sigma-Aldrich). Transiently transfected primary cortical neurons or SH-SY5Y cells were immunostained with combinations of rabbit polyclonal anti-βIII-tubulin antibody (Sigma-Aldrich), mouse monoclonal anti-V5 antibody (Invitrogen), or rabbit monoclonal anti-calreticulin antibody (EMD Millipore). Confocal images were acquired on a Zeiss LSM 700 confocal microscope using Zeiss ZEN confocal software (2009; Carl Zeiss AG, Feldbach, Switzerland) and a Plan-Apochromat 63x/1.40 oil objective in x, y and z planes. Image analysis was performed using Fiji software (Image Processing and Analysis in Java) or Imaris software (Bitplane AG, Zurich, Switzerland). Images were subjected to deconvolution using HuygensPro software (Scientific Volume Imaging, Hilversum, Netherlands). Pearson's correlation coefficients (Rcoloc) were calculated in Fiji between the indicated fluorescent channels. Representative images are taken from a single z-plane at a thickness of 0.15 µm.

### Cell viability assays

SH-SY5Y cells were seeded in growth medium in 96-well plates (25,000 cells/well) and transiently transfected with plasmid DNA. After 48 h, cellular viability was assessed by MTS reduction assay (CellTiter 96 Cell Proliferation Assay; Promega, Madison, WI, USA) according to manufacturer's instructions. Absorbance was measured on an Apollo-1 LB 911 microplate photometer (Berthold Technologies GmbH, Regensdorf, Switzerland) and background absorbance from media alone was subtracted. Viability was expressed as a percent of control (empty plasmid).

### Neurite outgrowth assays

Primary cortical cultures at DIV 3 were co-transfected with V5-ATP13A2 or empty vector and EGFP plasmids at a 10∶1 DNA molar ratio. At DIV 6, cultures were fixed with 4% PFA and subjected to immunocytochemistry with mouse anti-MAP2 antibody (Sigma-Aldrich) and anti-mouse IgG-AlexaFluor-633 antibody (Invitrogen). Where indicated primary cortical neurons were treated with cadmium (30 µM) or nickel (50 µM) for 24 h prior to fixation. Fluorescent images were acquired using an EVOS inverted fluorescence digital microscope (Advanced Microscopy Group, Bothell, WA, USA) with a 10x objective. EGFP images were pseudo-colored using ICA1 in NIH ImageJ software to improve the contrast of neuritic processes, and used for neurite length measurements. The length of EGFP^+^ neurites from MAP2^+^ cortical neurons were measured using the line tool function of NIH ImageJ software by an investigator blinded to each condition. Only neurons that had extended neurites were measured whereas neurons without processes were excluded from the analysis. The longest EGFP^+^ neurite (i.e. axon) was measured and used for comparison amongst groups. In each experiment, cortical neuronal processes from EGFP^+^/MAP^+^ neurons (*n* = 67–88) randomly sampled across five coverslips from at least three independent experiments were measured. For neurons treated with heavy metals, EGFP^+^/MAP^+^ neurites from control (*n* = 73–156), cadmium (*n* = 39–119) or nickel (*n* = 32–72) treatment groups were measured.

### Statistics

Data were analyzed by one-way ANOVA with Newman-Keuls post-hoc analysis as indicated. *P*<0.05 was considered significant.

## Supporting Information

Figure S1 **Analysis of human ATP13A2 variant mRNA expression levels.** ***A***, Agarose gel electrophoresis indicates similar DNA quantity and integrity for each ATP13A2 expression plasmid (500 ng DNA). Equivalent quantities of each plasmid were employed for transient transfection of HEK-293T cells. ***B***, Quantitative RT-PCR was conducted on mRNA-derived cDNAs from HEK-293T cells transiently expressing V5-tagged human ATP13A2 variants. PCR primers specific for plasmid-derived human ATP13A2 were employed that amplify the 3′ end of ATP13A2 incorporating the V5 epitope tag sequence. Bars represent the relative levels of plasmid-derived human ATP13A2 mRNA normalized to endogenous β-actin mRNA levels expressed in arbitrary units (mean±SEM, *n* = 4 independent experiments). Non-significant (*ns*) compared to WT ATP13A2 by one-way ANOVA with Newman-Keuls post-hoc analysis.
(TIF)
