# Supplementary figures and images for: Common Pathogenic Effects of Missense Mutations in the P-Type ATPase ATP13A2 (PARK9) Associated with Early-Onset Parkinsonism
Source: PLoS One. 2012 Jun 29;7(6):e39942. doi: 10.1371/journal.pone.0039942 (PMC3386943; doi:10.1371/journal.pone.0039942)

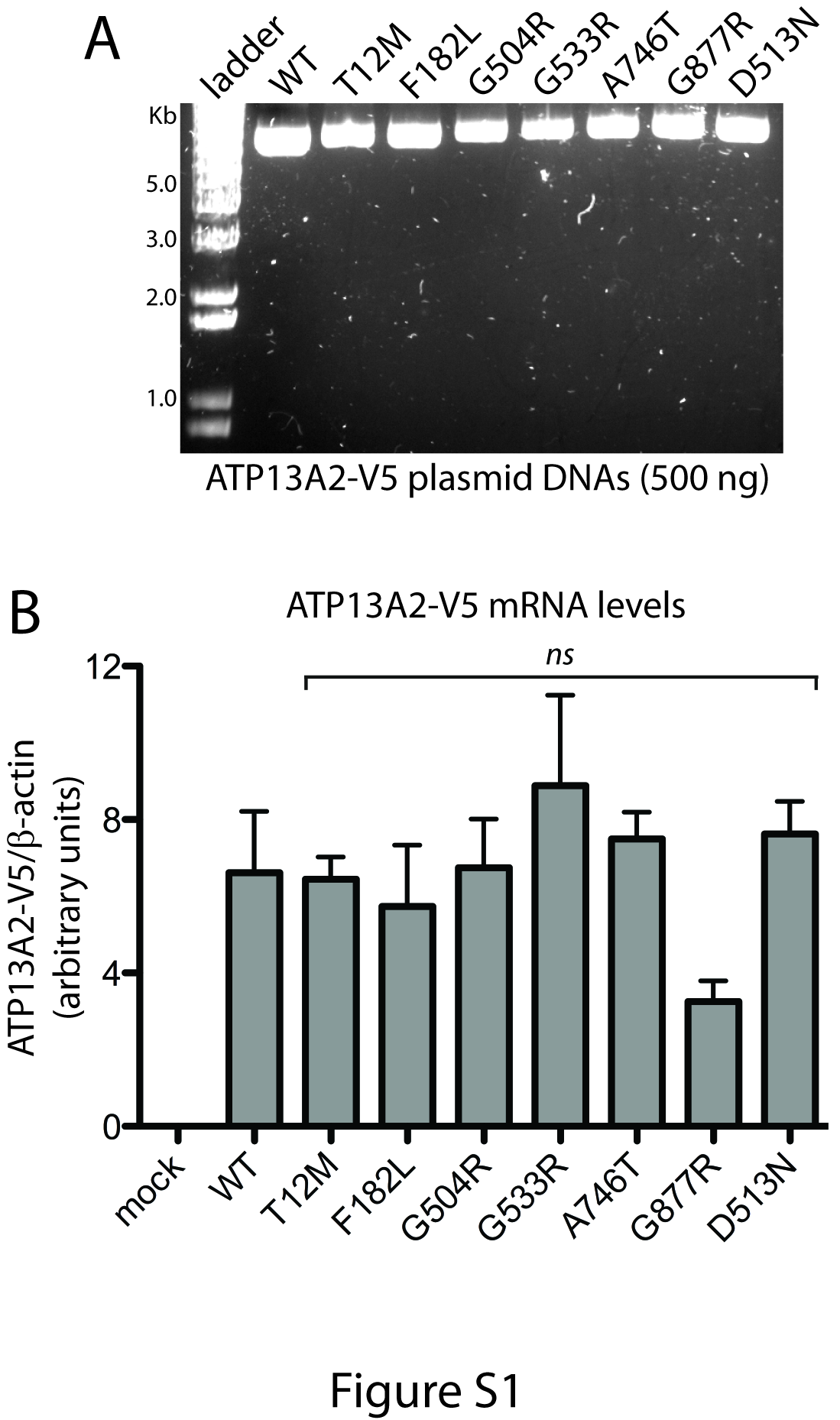

Supplement: Figure S1 — Analysis of human ATP13A2 variant mRNA expression levels. A, Agarose gel electrophoresis indicates similar DNA quantity and integrity for each ATP13A2 expression plasmid (500 ng DNA). Equivalent quantities of each plasmid were employed for transient transfection of HEK-293T cells. B, Quantitative RT-PCR was conducted on mRNA-derived cDNAs from HEK-293T cells transiently expressing V5-tagged human ATP13A2 variants. PCR primers specific for plasmid-derived human ATP13A2 were employed that amplify the 3′ end of ATP13A2 incorporating the V5 epitope tag sequence. Bars represent the relative levels of plasmid-derived human ATP13A2 mRNA normalized to endogenous β-actin mRNA levels expressed in arbitrary units (mean±SEM, n = 4 independent experiments). Non-significant (ns) compared to WT ATP13A2 by one-way ANOVA with Newman-Keuls post-hoc analysis. (TIF) [file pone.0039942.s001.tif]
